# Routine Application of a Bioabsorbable Cellulose Hemostatic Agent (WoundClot^TM^) in Unilateral Biportal Endoscopic Lumbar Decompression: A Retrospective Comparative Study

**DOI:** 10.3390/jcm15187279

**Published:** 2026-09-19

**Authors:** JinWoo Jung, Sang-Woo Lee, Donghyun Kim, Young San Ko, Dae-Chul Cho, Sang-Kyu Son, Man-Kyu Park

**Affiliations:** 1 Department of Neurosurgery, Hu Hospital, Busan 47261, Republic of Korea; jjweve@naver.com (J.J.);; 2 Department of Radiology, Seoul National University Hospital, Seoul National University College of Medicine, Seoul 03080, Republic of Korea; 3 Department of Neurosurgery, Kyungpook National University Hospital, School of Medicine, Kyungpook National University, Daegu 41405, Republic of Korea; 4 Department of Neurosurgery, Good Moonhwa Hospital, Busan 48735, Republic of Korea

**Keywords:** spinal stenosis, spinal epidural hematoma, hemostatics, endoscopic hemostasis

## Abstract

**Objective:** Unilateral biportal endoscopic (UBE) lumbar decompression is an effective minimally invasive spine surgery, but postoperative spinal epidural hematoma (PSEH) remains a practical concern. This study evaluated the clinical necessity of routine application of a bioabsorbable cellulose-based hemostatic agent in UBE lumbar decompression. **Methods:** This retrospective comparative study included 115 patients who underwent UBE unilateral laminectomy and bilateral decompression for lumbar spinal stenosis. Patients were divided into a control group without routine hemostatic agent application (*n* = 66) and a hemostatic group with routine hemostatic agent application (*n* = 49). Clinical outcomes, operation time, postoperative drain output, hemoglobin change, and PSEH on postoperative day (POD) 2 MRI were compared. **Results:** Baseline characteristics were comparable between the groups. Total drain output was significantly lower in the hemostatic group than in the control group (61.50 [50.00–78.50] mL vs. 77.00 [59.85–96.75] mL, *p* = 0.002), particularly on POD 2 (19.00 [11.20–22.50] mL vs. 25.00 [18.25–40.00] mL, *p* < 0.001). Operation time was also shorter in the hemostatic group (41.43 ± 6.69 min vs. 45.91 ± 6.32 min, *p* < 0.001). However, hemoglobin change, clinical outcomes, and PSEH incidence did not significantly differ between the groups. Symptomatic PSEH requiring revision surgery occurred in one control patient and in no hemostatic patients. **Conclusions:** Routine hemostatic agent application was not associated with a significant reduction in PSEH after UBE lumbar decompression. Although lower POD 2 drain output and shorter operative time were observed in the hemostatic group, the clinical significance of these findings remains uncertain and warrants further prospective study.

## 1. Introduction

Unilateral biportal endoscopic (UBE) lumbar decompression has emerged as an effective minimally invasive technique for the management of lumbar spinal stenosis. By utilizing continuous saline irrigation and independent scope and working portals, UBE provides magnified visualization while minimizing muscle and soft tissue injury. However, despite the theoretical tamponade effect of hydrostatic pressure, intraoperative bleeding and postoperative spinal epidural hematoma (PSEH) remain a practical challenge [1]. Even minor venous bleeding can compromise visualization in the confined endoscopic field and arterial bleeding can lead to catastrophic consequences such as PSEH [2].

Various topical hemostatic agents have been introduced in endoscopic spine surgery to improve bleeding control and surgical field clarity [3]. Among these, WoundClot^TM^ (Core Scientific Creations Ltd., Petah Tikva, Israel) is designed to form a gel-like matrix upon contact with blood, facilitating local clot stabilization without relying on thrombin-mediated coagulation activation. While such agents are widely used in various open surgeries and wound dressings, their application in UBE lumbar decompression remains controversial [4,5,6]. Continuous saline irrigation in UBE already provides partial hemostasis, and it is unclear whether additional topical hemostatic augmentation offers clinically meaningful benefit.

Therefore, this study aimed to evaluate the clinical necessity of the routine application of a bioabsorbable cellulose hemostatic agent in patients undergoing UBE lumbar decompression. Specifically, we compared postoperative drain output and hematoma incidence between cases performed with and without adjunctive hemostatic material. To the best of our knowledge, no comparative study has examined the use of a cellulose-based hemostatic agent in UBE lumbar decompression, and this study represents the first analysis addressing its impact on postoperative care.

## 2. Materials and Methods

### 2.1. Patient Enrollment

This retrospective comparative cohort study enrolled 115 consecutive patients who underwent UBE-unilateral laminectomy bilateral decompression (ULBD) for lumbar spinal stenosis at our institution between July 2024 and April 2026. This study protocol was approved by the Institutional Review Board of the Korea National Institute for Bioethics Policy (P01-202605-01-041).

**Figure 1:**
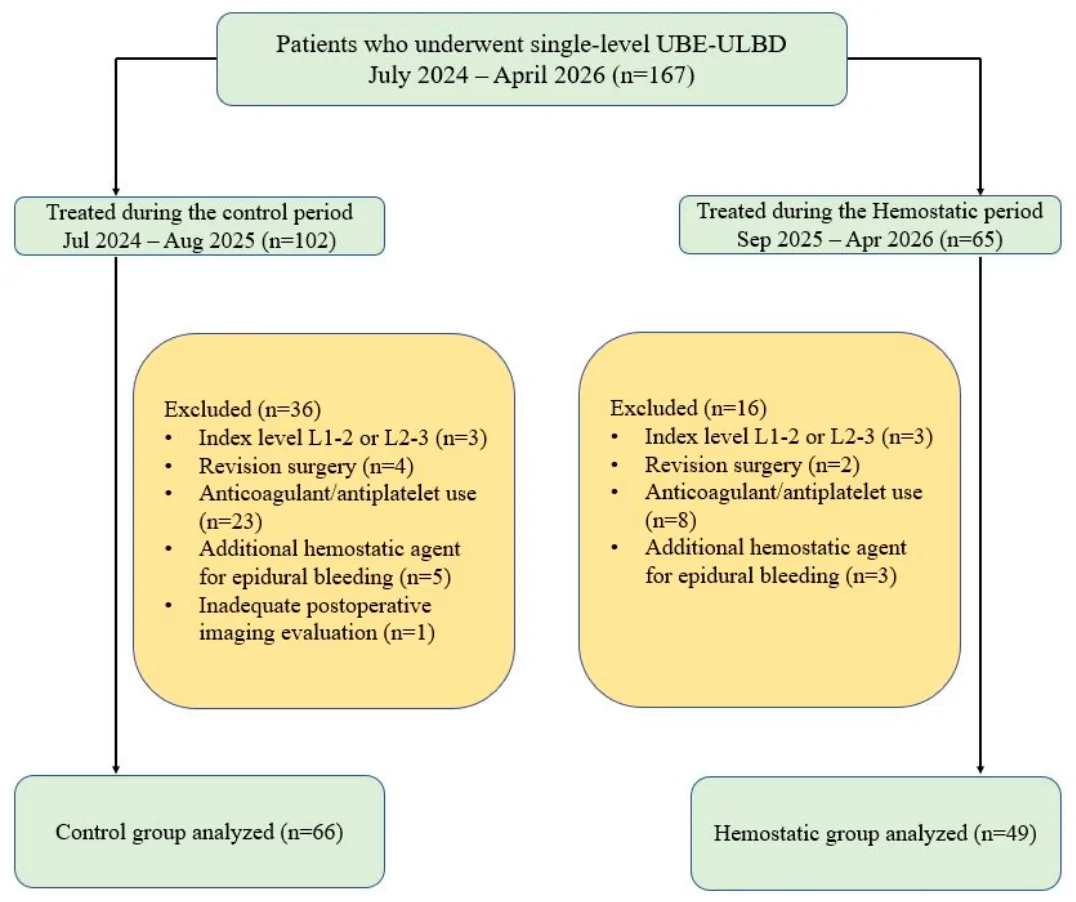
Participant flow diagram according to the STROBE recommendations. STROBE: Strengthening the Reporting of Observational Studies in Epidemiology, UBE: Unilateral biportal endoscopy, ULBD: Unilateral laminectomy bilateral decompression.

Patients were divided into two groups according to the routine application of a bioabsorbable cellulose hemostatic agent (Figure 1). From July 2024 to August 2025, ULBD was performed without routine use of the hemostatic agent (Control group, *n* = 66). From September 2025 to April 2026, ULBD was performed with routine use of the hemostatic agent (Hemostatic group, *n* = 49). All procedures were performed by a single expert spine surgeon who had performed more than 5000 UBE cases. No formal changes were made to the UBE ULBD surgical technique or the postoperative management protocol between the two treatment periods.

The inclusion criteria were as follows: (1) age ≥ 18 years, (2) bilateral lumbar radiculopathy and/or neurogenic intermittent claudication, (3) clinical symptoms consistent with magnetic resonance imaging (MRI) findings of lumbar central stenosis, (4) single-level lumbar ULBD and (5) failure of at least 6 weeks of conservative treatment or progressive motor deficit. Patients were excluded as follows: (1) ULBD performed at the L1-2 or L2-3 level, (2) unilateral lumbar radiculopathy, (3) segmental instability requiring fusion surgery, (4) concomitant foraminal stenosis, (5) previous lumbar spine surgery at the same segment, (6) concurrent infection, trauma, or tumor, (7) Any use of antiplatelet or anticoagulant medication for underlying cardiovascular or cerebrovascular disease within the preoperative period, (8) intraoperative use of additional hemostatic agents, or (9) inadequate preoperative and postoperative imaging evaluation.

### 2.2. Surgical Technique

All procedures were performed under general anesthesia with the patient in the prone position. To minimize intra-abdominal pressure and reduce epidural venous bleeding, the abdomen was allowed to hang freely using chest and pelvis sponges. The surgical level was confirmed using intraoperative C-arm fluoroscopy after standard sterile preparation and draping.

Two small transverse skin incisions (approximately 0.5–1 cm) were made at the medial margin of the pedicle. Following fascial incision, serial dilators were introduced into the interfascial plane of the lumbar multifidus muscle to establish the working space. A 0° endoscope was inserted through the scope portal, and continuous saline irrigation was maintained with adequate outflow through the working portal.

**Figure 2:**
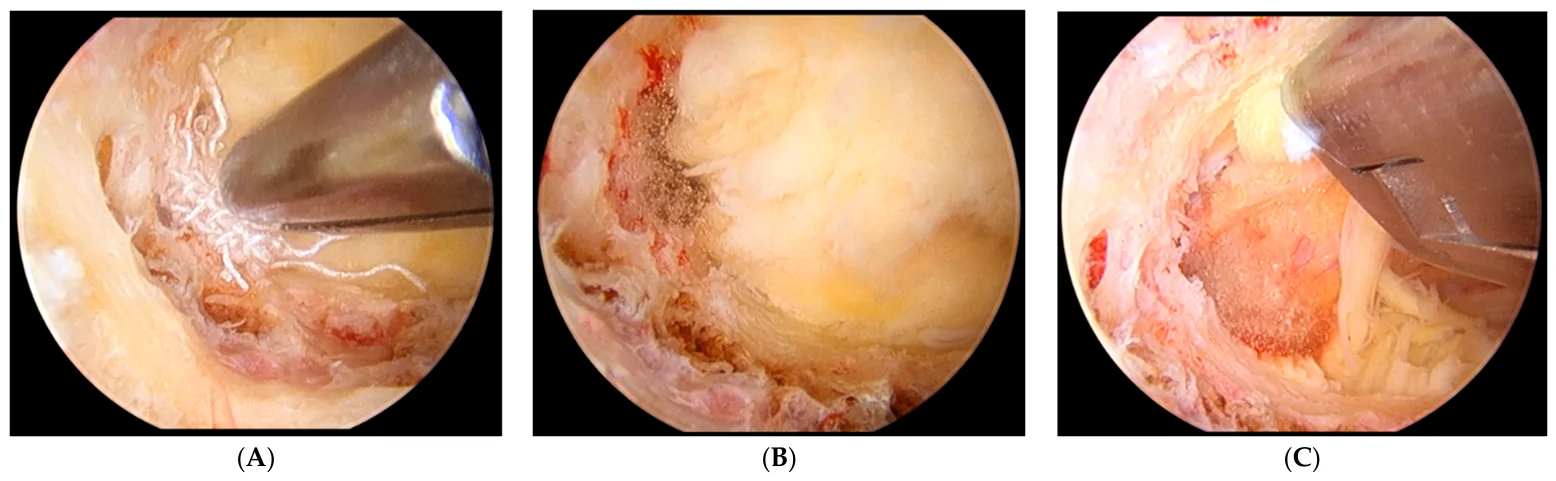
Serial intraoperative endoscopic images after application of bioabsorbable cellulose-based hemostatic materials at the end of the ligamentum flavum. (**A**) Immediately after application. (**B**) Ten minutes after application. (**C**) Thirty minutes after application.

The initial docking point was the spinolaminar junction. Ipsilateral partial laminectomy was first performed to expose the ligamentum flavum (LF), and the midline cleft of the LF was identified as a key anatomical landmark. Drilling was extended cranially to fully expose the cranial detachment of the LF. Because prominent epidural vessels are frequently encountered around the cranial end of the LF, bioabsorbable cellulose-based hemostatic material was packed in this area in the hemostatic group (Figure 2). For contralateral decompression, the contralateral LF was carefully detached using a Freer elevator, and a sublaminar drilling was established. Contralateral laminectomy was then performed until the medial border of the contralateral facet joint was visualized.

After completion of contralateral bone working, the ipsilateral side was revisited to identify the ipsilateral caudal lamina. Using a Freer elevator, a portion of the superficial layer of LF was removed from the upper portion of the caudal lamina. Partial resection of the caudal lamina and medial part of the superior articular process (SAP) was required to remove the deep layer of LF. After exposing the lateral margin of the thecal sac and the traversing root, sufficient foraminotomy was performed along the course of the nerve root. In cases with concomitant disc herniation, additional discectomy and annulotomy were performed as needed.

Subsequently, contralateral decompression was completed in a similar manner. The superficial and deep layers of the LF were removed from the contralateral caudal lamina, and the traversing nerve root was identified and decompressed. Bilateral decompression was confirmed by ensuring free mobilization of both traversing nerve roots.

**Figure 3:**
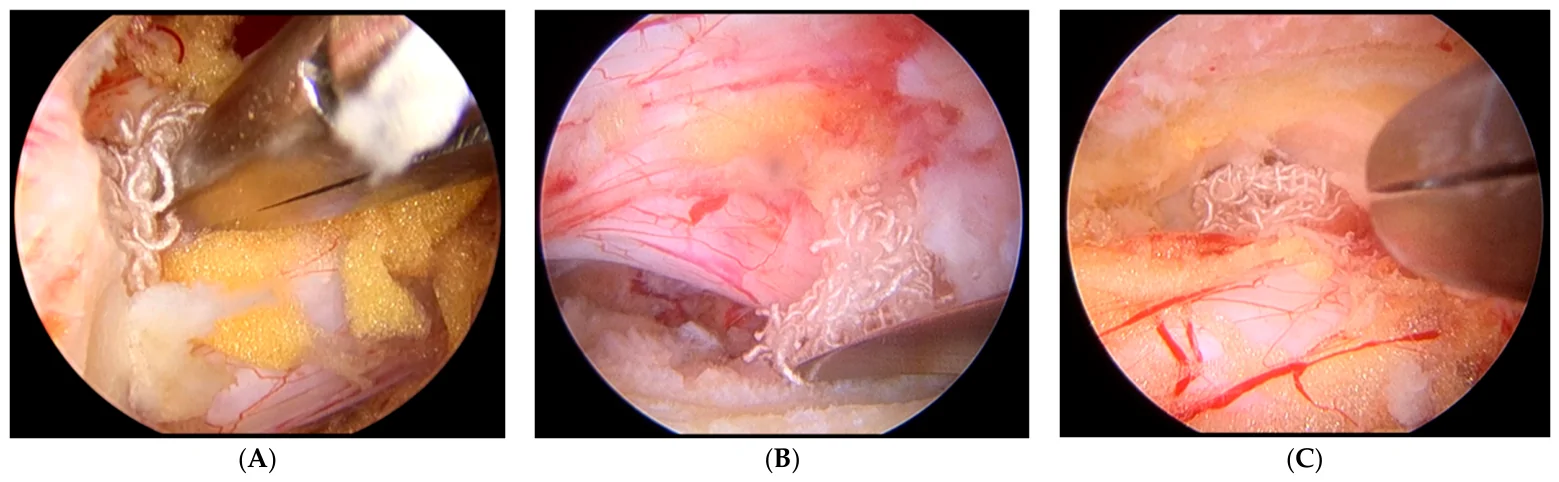
Final hemostatic agent packing after completion of unilateral biportal endoscopic unilateral laminectomy bilateral decompression. (**A**) Packing at the cranial drilling margin. (**B**) Packing beneath the left traversing nerve root. (**C**) Packing beneath the right traversing nerve root.

Meticulous bleeding control was achieved throughout the procedure. The most common sources of bleeding during ULBD were the epidural venous plexus around both traversing nerve roots and the cranial attachment of the LF. Arterial bleeding was controlled using radiofrequency (RF) coagulation, and osseous bleeding was managed with bone wax in all cases. In the hemostatic group, bioabsorbable cellulose-based hemostatic materials were additionally applied to control epidural venous bleeding, specifically beneath both traversing nerve roots and at the cranial drilling margin (Figure 3). The hemostatic agent was applied according to a fixed protocol in all patients in the hemostatic group. A single 5 × 5 cm sheet of the bioabsorbable cellulose hemostatic agent was cut into pieces of approximately 1 × 1 cm. The same fixed amount was used in every patient in the hemostatic group, and no additional material was applied regardless of the degree of intraoperative venous oozing. All applied pieces were left in situ at wound closure. A Jackson-Pratt (J-P) surgical drain (100 cc) was placed, and the wound was closed layer by layer.

### 2.3. Preoperative and Postoperative Management Protocol

All patients underwent standardized preoperative and postoperative management protocols. Preoperatively, patients were treated with at least 6 weeks of conservative treatment, including oral medications and interventional procedures such as epidural or selective nerve root block. Preoperative imaging included lumbar spine radiographs (anteroposterior, lateral, flexion and extension views), computed tomography (CT), and MRI to evaluate the extent of spinal stenosis. Routine laboratory evaluation was performed on the day before surgery, including a complete blood count (CBC) to assess baseline hemoglobin (Hb) and platelet levels, and a coagulation panel from which the international normalized ratio (INR) was obtained as a measure of baseline hemostatic competence.

Postoperatively, early ambulation was encouraged immediately after surgery in all patients. A J-P surgical drain was routinely placed and maintained until postoperative day (POD) 2. Drain output was recorded on POD 1 and POD 2. Hb levels were measured using CBC on POD 2. On POD 2, all patients underwent follow-up imaging, including lumbar spine radiographs (anteroposterior and lateral views), CT and MRI. After the POD 2 MRI was obtained, the drain was removed in all patients irrespective of the radiographic findings.

### 2.4. Clinical and Radiological Measurements

Demographic and clinical data were collected, including sex, age, body mass index (BMI), surgical level, operation time, and length of hospital stay. Operation time was defined as the interval from skin incision to completion of skin closure, excluding anesthesia induction and patient positioning. Comorbidities relevant to bleeding risk were recorded as the presence or absence of hypertension and diabetes mellitus, based on a documented diagnosis or ongoing pharmacological treatment at the time of admission. Procedure-related complications were also recorded. Clinical outcomes were assessed using the visual analog scale (VAS) for both back pain and leg pain. Pain intensity was evaluated preoperatively and on POD 3 to reflect early postoperative clinical status.

**Figure 4:**
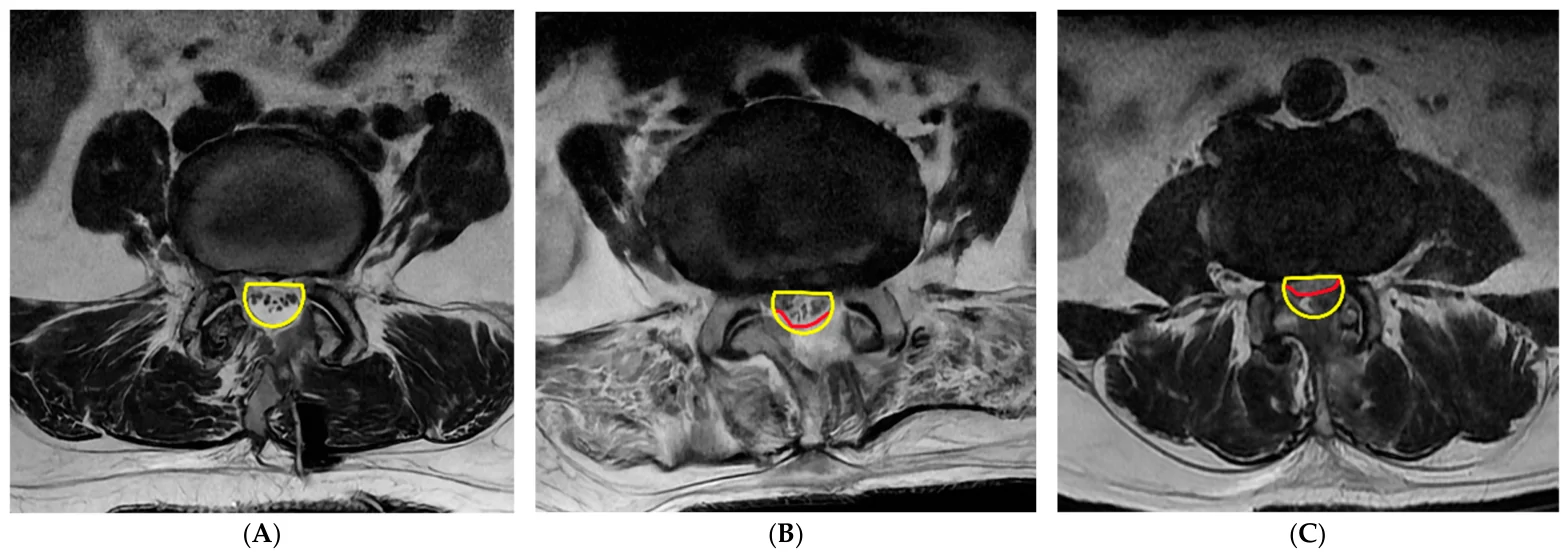
Postoperative spinal epidural hematoma grading system on axial T2-weighted MRI. (**A**) Grade 0, no hematoma. (**B**) Grade I, hematoma causing <25% compression of the spinal canal. (**C**) Grade III, hematoma causing 50–75% compression of the spinal canal. The yellow outlines indicate the margins of the thecal sac, and the red lines delineate the boundary between the hematoma and the thecal sac.

Radiological measurements focused on the presence of PSEH using MRI obtained on POD 2. Preoperative and POD 2 MRI were obtained on a 1.5-T scanner (GE Healthcare, Milwaukee, WI, USA) with a slice thickness of 3 mm. Preoperative MRI consisted of T1- and T2-weighted sagittal and axial images. POD 2 MRI consisted of only T2-weighted sagittal and axial images. All MRI images were graded by a single board-certified radiologist who had no access to the electronic medical records and was not informed of the study design, the treatment-period cut-off, or whether a hemostatic agent had been applied. The reader was therefore blinded to treatment group, calendar period, and clinical symptoms. The MRI appearance of the bioabsorbable cellulose-based hemostatic agent has not been characterized in the previous literature, and no product-specific reader training was performed. PSEH was defined as a collection causing compression of the dural sac on T2 axial images. The degree of dural sac compression was evaluated at the level of maximal hematoma and categorized into five grades: grade 0, no hematoma; grade I, <25% compression of the spinal canal; grade II, 25–50% compression; grade III, 50–75% compression; and grade IV, >75% compression (Figure 4) [2].

PSEH was further classified as symptomatic or asymptomatic based on clinical correlation. Hematomas were considered symptomatic when associated with persistent or aggravated postoperative symptoms. In contrast, hematomas in patients whose symptoms improved or remained unchanged relative to the preoperative status were classified as asymptomatic. Revision surgery for PSEH was indicated when a patient with symptomatic PSEH developed a progressive motor deficit or cauda equina syndrome, or when aggravated back or radicular pain was refractory to conservative treatment, including steroid injection and opioid analgesics.

### 2.5. Outcome Measurements

The primary outcome of this study was the incidence of radiological PSEH causing compression of the dural sac (grade I or higher) on T2-weighted axial MRI obtained on POD 2. This outcome was designated as primary in accordance with the stated study objective. However, owing to the retrospective design, it was not prospectively registered. Secondary outcomes included the incidence of symptomatic PSEH, PSEH grade, total and daily postoperative drain output, operation time, hemoglobin change, and back and leg VAS scores. Drain output and operation time were regarded as mechanistic or surrogate measures of hemostatic efficacy rather than direct measures of clinical benefit. Hb change was reported as a descriptive perioperative variable.

### 2.6. Statistical Analysis

All statistical analyses were performed using R software version 4.3.0 (R Foundation for Statistical Computing, Vienna, Austria) by an independent statistician. The distribution of continuous variables was assessed using the Shapiro–Wilk test. Normally distributed variables were calculated as mean ± standard deviation and analyzed using an independent *t*-test. Non-normally distributed variables were presented as median with interquartile range and compared using the Mann–Whitney U test. Categorical variables were calculated as frequencies with percentages and compared using the chi-square test or Fisher’s exact test, as appropriate. For the primary and secondary PSEH outcomes, absolute event rates were reported for each group, and absolute risk differences with 95% confidence intervals (CIs) were calculated using the Newcombe hybrid score method. Relative risks with 95% CIs were estimated when statistically estimable. Relative estimates were not calculated when no events occurred in either group. Each *p* values from Fisher’s exact test were reported for sparse event data. No formal sample size calculation was performed before the analysis. Instead, all consecutive eligible patients treated during the study period were included. A *p* value <0.05 was considered statistically significant.

## 3. Results

### 3.1. Patient Demographics and Perioperative Characteristics

Patient demographics and perioperative characteristics are summarized in Table 1. A total of 115 patients were included in this study, with 66 patients in the control group and 49 patients in the hemostatic group. There were no significant differences between the two groups in age, sex, surgical level or diagnosis. Preoperative Hb, POD 2 Hb, and change in Hb level were also comparable between the groups.

The total J-P surgical drain output was significantly lower in the hemostatic group than in the control group (61.50 [50.00–78.50] mL vs. 77.00 [59.85–96.75] mL, *p* = 0.002). In particular, POD 2 drain output was markedly reduced in the hemostatic group (19.00 [11.20–22.50] mL vs. 25.00 [18.25–40.00] mL, *p* < 0.001), whereas POD 1 drain output did not significantly differ between the groups. Operation time was significantly shorter in the hemostatic group than in the control group (41.43 ± 6.69 min vs. 45.91 ± 6.32 min, *p* < 0.001). Length of hospital stay and complication rates were not significantly different between the groups. A sensitivity analysis excluding the single control patient in whom Tachosil was applied for an incidental durotomy did not alter these findings.

**Figure 5:**
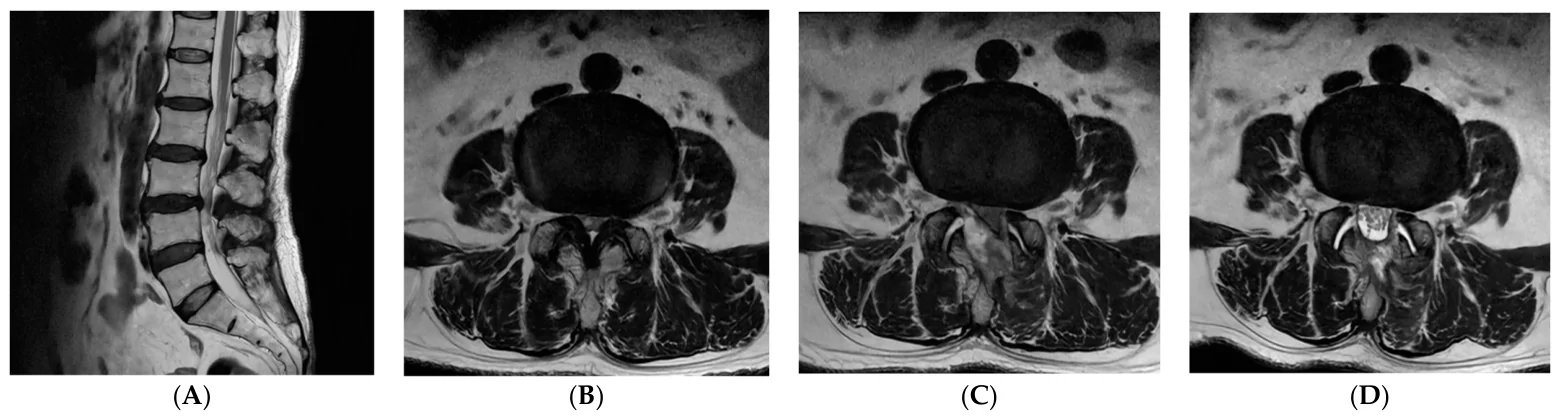
Symptomatic postoperative spinal epidural hematoma (PSEH) after L3-4 unilateral biportal endoscopic lumbar decompression. A 79-year-old female patient underwent L3-4 unilateral laminectomy and bilateral decompression for central stenosis and bilateral lateral recess stenosis. (**A**) Preoperative T2-weighted sagittal MRI. (**B**) Preoperative T2-weighted axial MRI. (**C**) T2-weighted axial MRI on postoperative day 2 showing grade 3 PSEH. (**D**) T2-weighted axial MRI after revision surgery.

Two complications occurred in the control group. One patient had a small intraoperative dural tear, which was managed with TachoSil (Corza Medical, Westwood, MA, USA) application in the surgical field. In this patient, Tachosil was applied solely as a dural sealant to repair the incidental durotomy and was not used for the purpose of hemostasis. The patient remained on bed rest until POD 2 and was discharged on POD 6 without further complications. The other patient developed symptomatic PSEH with aggravated back pain and radiculopathy compared with the preoperative status. As the pain was refractory to conservative management, revision surgery for hematoma evacuation was performed on POD 2, after which the symptoms improved. The patient was discharged on POD 7 without further events (Figure 5).

### 3.2. Clinical and Radiological Outcomes

Clinical and radiological outcomes are summarized in Table 2. Preoperative back VAS and leg VAS were comparable between the control and hemostatic groups. At POD 3, both back and leg VAS showed no significant differences between the two groups.

Radiological evaluation of PSEH on POD 2 MRI also demonstrated no significant difference between the groups. Most patients showed no postoperative hematoma in both groups. Radiological PSEH was observed in 3 patients in the control group and 2 patients in the hemostatic group. The distribution of PSEH grades was not significantly different between the two groups. Regarding clinical manifestation, symptomatic PSEH occurred in 1 patient in the control group and no patients in the hemostatic group. The incidence of symptomatic or asymptomatic PSEH was not significantly different between the groups.

For the primary outcome, radiographic PSEH on POD 2 MRI was observed in 3 of 66 patients (4.55%) in the control group and 2 of 49 patients (4.08%) in the hemostatic group (absolute risk difference −0.46 percentage points, relative risk 0.90, *p* = 1.000) (Table 3). Symptomatic PSEH occurred in 1 of 66 patients (1.52%) in the control group and in none of the 49 patients (0.00%) in the hemostatic group (absolute risk difference −1.52 percentage points, *p* = 1.000). A relative risk could not be estimated because no events occurred in the hemostatic group.

## 4. Discussion

Topical hemostatic materials have been widely utilized in surgical practice to facilitate bleeding control and improve intraoperative surgical view. Among these, cellulose-based hemostatic agents were originally developed for use in trauma and prehospital settings, where rapid control of external bleeding is critical [5]. WoundClot^TM^, a bioabsorbable cellulose-based hemostatic agent, represents a next-generation development. Although such materials have demonstrated effectiveness in managing external bleeding and in various surgical fields, their role in endoscopic spine surgery, particularly in UBE lumbar decompression, has not been clearly established [4,6,7,8].

PSEH is a serious complication following spine surgery [9,10,11]. When patients develop postoperative neurological deficits in the presence of radiologically confirmed PSEH, urgent surgical intervention may be required. To minimize risk of PSEH, meticulous intraoperative hemostasis is essential. Standard hemostatic strategies include adequate coagulation of bleeding vessels and control of osseous bleeding using bone wax [12,13]. These principles are equally applicable in UBE surgery. Although UBE is minimally invasive spine surgery associated with reduced muscle and soft tissue injury, the resulting limited epidural space may increase the susceptibility of neural elements to even small-volume hematoma [14]. In such a confined space, minimal postoperative bleeding may lead to clinically relevant dural sac or nerve root compression. Based on these considerations, our institutional protocol includes routine placement of a J-P drain until POD 2.

Our findings should be interpreted in the context of previous studies of other topical hemostatic agents in endoscopic spine surgery. In a retrospective case–control study of UBE decompression, Ko et al. reported that routine use of a thrombin-containing local hemostatic was associated with a significantly lower proportion of severe radiographic hematoma [15]. However, the difference in symptomatic PSEH did not reach statistical significance. Kim et al. similarly reported that a gelatin-thrombin matrix sealant reduced the incidence of radiographic PSEH and prevented high-grade hematoma in UBE [16]. In a randomized trial of 96 patients undergoing UBE decompression, Yan et al. found that flowable gelatin matrix reduced perioperative bleeding, and symptomatic PSEH occurred only in the control group [17]. In contrast, in a randomized trial of microendoscopic laminotomy, Takami et al. found that prophylactic use of a gelatin-thrombin matrix sealant did not reduce postoperative drainage volume or hematoma formation, and the authors advised against its routine prophylactic use [18].

In the present study, no significant differences were observed between the two groups in clinical or radiological outcomes. The incidence of radiological and symptomatic PSEH was not statistically different between the groups. Nevertheless, one patient in the control group developed symptomatic PSEH requiring revision surgery, whereas no symptomatic PSEH occurred in the hemostatic group. Moreover, PSEH cases in the hemostatic group were all asymptomatic and limited to grade 1 PSEH, while the control group included radiologically more severe PSEH cases. These findings should be interpreted cautiously because of the small number of PSEH events and retrospective study design. However, the possibility that hemostatic agent may reduce the severity of PSEH or lower the risk of symptomatic progression cannot be excluded. Further prospective randomized trials with large sample sizes are warranted to clarify the preventive effect of the hemostatic agent on clinically relevant PSEH after UBE lumbar decompression.

Although operation time was statistically shorter in the hemostatic groups, the absolute mean difference between the groups was only 4.48 min. Therefore, the clinical relevance of this reduction at the individual patient level is likely limited. In addition, as noted in the Methods, the two groups were defined according to consecutive treatment periods. Although no formal changes were made to the surgical technique or postoperative protocol, temporal improvements in operative workflow, surgical team coordination, or other unmeasured factors may have contributed to the observed difference. This possibility cannot be completely excluded, even though all procedures were performed by a highly experienced spine surgeon with more than 5000 UBE cases. Accordingly, shorter operation time should not be interpreted as evidence of a clinically meaningful benefit or definitive superiority of the hemostatic agent. Despite the limited clinical magnitude of the reduction in operation time, the observed difference may provide indirect insight into the intraoperative effects of the hemostatic agent. One possible explanation is improved intraoperative endoscopic visualization resulting from reduced epidural venous oozing. Improved visualization may facilitate more efficient decompression and shorten operation time. Previous studies have attempted to quantify intraoperative endoscopic visualization using specialized image analysis tools or grading systems [19,20,21]. However, objective assessment of endoscopic field clarity remains challenging. Therefore, although the observed reduction in operation time may indirectly reflect improved visualization, further studies incorporating objective and reproducible metrics are needed to validate this effect.

The change in Hb levels did not significantly differ between the two groups. However, perioperative intravenous fluid volume was not available from the retrospective records, and hemodilution may therefore have influenced the measured Hb change. For this reason, we did not interpret Hb change as a surrogate for perioperative blood loss. Another notable finding was the significant reduction in drain output on POD 2 in the hemostatic group. In UBE surgery, drain output on POD 1 represents a mixture of residual saline irrigation fluid and postoperative bleeding. In contrast, drain output on POD 2 is less influenced by saline irrigation fluid and more reflective of postoperative bleeding. Therefore, the observed reduction in drain output on POD 2 suggests that the application of the hemostatic agent may effectively reduce epidural venous oozing following surgery. However, postoperative drain output is an indirect surrogate for surgical site bleeding and may also be influenced by drain position, patency, and other patient-related factors. Moreover, because routine postoperative drainage was used in all patients, the present study could not determine whether hemostatic agent application could safely eliminate the need for postoperative drainage. Further prospective studies are required to determine whether the reduction in drain output translates into clinically meaningful benefits.

The exact bleeding source of PSEH remains controversial. Studies of spontaneous spinal epidural hematoma have suggested that both venous and arterial bleeding are possible [22]. However, rapidly progressive and clinically relevant epidural hematoma is more likely to be related to inadequately controlled active arterial bleeding rather than diffuse venous oozing [23]. These reports concern spontaneous hematoma, however, and the bleeding source of hematoma occurring after UBE decompression has not been established. The present study likewise provides no information on the bleeding source, as this was not assessed. Nonetheless, from a practical standpoint, definite epidural arterial bleeding encountered during UBE surgery should be meticulously controlled using RF coagulation rather than relying solely on topical hemostatic materials. Epidural venous oozing during UBE surgery may be partially masked by continuous saline irrigation and hydrostatic pressure. Technical reviews of UBE have noted that hydrostatic pressure from the irrigation system can suppress low-pressure venous bleeding or cancellous bone bleeding [24,25]. However, once irrigation is reduced or stopped at the end of the surgery, rebound oozing from the epidural venous plexus or cancellous bone may become apparent. In this situation, a hemostatic agent may be beneficial as an adjunct for epidural venous oozing, but not as a replacement for definite hemostasis of active arterial bleeding.

This study has several limitations. First, this was a retrospective, non-randomized study from a single institution. Because the use of the hemostatic agent was determined by the chronological treatment period rather than randomized controlled, selection bias and temporal bias cannot be excluded. Second, the sample size was relatively small, particularly in the hemostatic group. Therefore, this study may have been underpowered to determine the preventive effect of the hemostatic agent against clinically significant PSEH. Third, intraoperative endoscopic visualization was not objectively quantified. Although shorter operation time in the hemostatic group may indirectly reflect improved surgical field clarity, further studies using objective and reproducible assessment methods are required. Fourth, the evaluation of clinical and radiological outcomes was strictly limited to the early postoperative period. The potential long-term complications associated with leaving the hemostatic agent were not investigated in this study. Fifth, the MRI appearance of the bioabsorbable cellulose-based hemostatic agent at 48 h has not been systematically described, and the radiologist received no product-specific training [26]. According to the manufacturer, the material is absorbed within 7 to 14 days, so residual material could theoretically have been present at the time of POD 2 MRI. However, any residual material misinterpreted as hematoma would have inflated the PSEH rate in the hemostatic group. Finally, the routine application of adjunctive hemostatic materials inevitably increases the overall surgical cost. Given that the routine use of the hemostatic agent did not demonstrate a significant benefit in preventing PSEH, a comprehensive cost-effectiveness analysis is necessary to justify its routine clinical application.

## 5. Conclusions

In UBE lumbar decompression, the routine use of a bioabsorbable cellulose-based hemostatic agent is not associated with a statistically significant reduction in PSEH. Although lower POD 2 drain output and shorter operative time were observed in the hemostatic group, the clinical significance of these findings remains uncertain. Further prospective studies are required to clarify whether the hemostatic agent provides meaningful benefits.

## Figures and Tables

**Table 1:** Patient demographics and perioperative characteristics.

Characteristics	Control Group, *n* = 66	Hemostatic Group, *n* = 49	*p*
Age, years	63.55 ± 14.39	65.10 ± 11.01	0.512
Sex, M/F	35 (53.03%)/31 (46.97%)	23 (46.94%)/26 (53.06%)	0.518
BMI	24.73 ± 3.35	24.31 ± 3.11	0.489
HTN, *n* (%)	28 (42.42%)	23 (46.94%)	0.630
DM, *n* (%)	20 (30.30%)	12 (24.49%)	0.492
Surgical level			0.561
L3-4	16 (24.24%)	8 (16.33%)	
L4-5	46 (69.70%)	37 (75.51%)	
L5-S1	4 (6.06%)	4 (8.16%)	
Diagnosis			0.483
Spinal stenosis only	32 (48.48%)	27 (55.10%)	
Spinal stenosis with HNP	34 (51.52%)	22 (44.90%)	
Hemoglobin (g/dL)			
Preoperative Hb	13.65 ± 1.20	13.82 ± 1.42	0.509
POD2 Hb	12.04 ± 1.26	12.29 ± 1.34	0.319
Change in Hb level	−1.61 ± 0.74	−1.53 ± 0.61	0.520
Platelet count (×10^3^/μL)	240.15 ± 61.49	248.00 ± 71.74	0.530
INR	1.00 ± 0.07	1.020 ± 0.064	0.066
J-P surgical drain output (mL) ^†^			
POD 1	51.00 [37.50–62.50]	42.00 [37.00–55.00]	0.096
POD 2	25.00 [18.25–40.00]	19.00 [11.20–22.50]	<0.001 **
Total	77.00 [59.85–96.75]	61.50 [50.00–78.50]	0.002 **
Operation time (min)	45.91 ± 6.32	41.43 ± 6.69	<0.001 **
Length of hospital stay ^†^	7.00 [6.00–8.00]	6.00 [5.00–8.00]	0.279
Complications	2 (3.03%)	0 (0.00%)	0.507

HNP: herniated nucleus pulposus, Hb: hemoglobin, POD: postoperative day, INR: international normalized ratio, J-P: Jackson-Pratt, **: *p* < 0.01. ^†^: values are presented as median [interquartile range], and *p*-values were calculated using the Mann–Whitney U test.

**Table 2:** Comparison of clinical and radiological outcome variables between two groups.

Variable	Control Group	Hemostatic Group	*p*
Back VAS			
Preoperative	5.88 ± 1.78	5.37 ± 1.65	0.116
POD 3	3.08 ± 1.09	2.98 ± 0.66	0.558
Leg VAS			
Preoperative	7.58 ± 0.70	7.53 ± 0.58	0.707
POD 3	2.82 ± 0.91	2.49 ± 1.47	0.173
PSEH			
Radiological grade			0.497 ^†^
Grade 0 (no hematoma)	63 (95.45%)	47 (95.92%)	
Grade I (<25%)	1 (1.52%)	2 (4.08%)	
Grade II (25–50%)	0 (0.00%)	0 (0.00%)	
Grade III (50–75%)	2 (3.03%)	0 (0.00%)	
Grade IV (>75%)	0 (0.00%)	0 (0.00%)	
Clinical manifestation			1.000 ^†^
No hematoma	63 (95.45%)	47 (95.92%)	
Symptomatic	1 (1.52%)	0 (0.00%)	
Asymptomatic	2 (3.03%)	2 (4.08%)	

VAS: visual analog scale, POD: postoperative day, PSEH: postoperative spinal epidural hematoma. ^†^: *p* values were calculated using Fisher’s exact test owing to sparse event counts.

**Table 3:** Effect estimates for the primary and secondary PSEH outcomes.

Outcome	Control Group	Hemostatic Group	Risk Difference, pp (95% CI)	Relative Risk (95% CI)	*p* ^†^
Radiographic PSEH (grade ≥ I)	3 (4.55%)	2 (4.08%)	−0.46 (−8.98~9.62)	0.90 (0.16~5.17)	1.000
Symptomatic PSEH	1 (1.52%)	0 (0.00%)	−1.52 (−8.10~5.86)	Not estimable ^‡^	1.000

PSEH: postoperative spinal epidural hematoma, pp: percentage points, CI: confidence interval, ^†^: *p* values were calculated using Fisher’s exact test owing to sparse event counts. ^‡^: Not estimable because no events occurred in the hemostatic group.

## Data Availability

The data presented in this study are not publicly available due to privacy and ethical restrictions. Data may be available from the corresponding author upon reasonable request, subject to approval by the Institutional Review Board.

## Abbreviations

The following abbreviations are used in this manuscript:

UBE	Unilateral biportal endoscopy
PSEH	Postoperative spinal epidural hematoma
ULBD	Unilateral laminectomy bilateral decompression
MRI	Magnetic resonance imaging
LF	Ligamentum flavum
SAP	Superior articular process
RF	radiofrequency
J-P	Jackson-Pratt
CT	Computed tomography
CBC	Complete blood count
Hb	Hemoglobin
POD	Postoperative day
BMI	Body mass index
INR	International normalized ratio
VAS	Visual analog scale
CI	Confidence interval
HNP	Herniated nucleus pulposus
